# A Mobile Text Message Intervention to Reduce Repeat Suicidal Episodes: Design and Development of Reconnecting After a Suicide Attempt (RAFT)

**DOI:** 10.2196/mental.7500

**Published:** 2017-12-13

**Authors:** Mark Erik Larsen, Fiona Shand, Kirsten Morley, Philip J Batterham, Katherine Petrie, Bill Reda, Sofian Berrouiguet, Paul S Haber, Gregory Carter, Helen Christensen

**Affiliations:** ^1^ Black Dog Institute University of New South Wales Sydney Australia; ^2^ National Health and Medical Research Council Centre of Research Excellence in Mental Health and Substance Use Discipline of Addiction Medicine University of Sydney Sydney Australia; ^3^ Centre for Mental Health Research Australian National University Canberra Australia; ^4^ Brest Medical University Hospital at Bohars, Adult Psychiatry IMT Atlantique, Lab-STICC, UBL, F-29238 Brest, France EA 7479 SPURBO, Université de Bretagne Occidentale Brest France; ^5^ Drug Health Services Royal Prince Alfred Hospital Sydney Australia; ^6^ Centre for Brain and Mental Health Research University of Newcastle Newcastle Australia

**Keywords:** suicide, attempted, emergency service, hospital, continuity of patient care, text messaging, Internet

## Abstract

**Background:**

Suicide is a leading cause of death, particularly among young people. Continuity of care following discharge from hospital is critical, yet this is a time when individuals often lose contact with health care services. Offline brief contact interventions following a suicide attempt can reduce the number of repeat attempts, and text message (short message service, SMS) interventions are currently being evaluated.

**Objective:**

The aim of this study was to extend postattempt caring contacts by designing a brief Web-based intervention targeting proximal risk factors and the needs of this population during the postattempt period. This paper details the development process and describes the realized system.

**Methods:**

To inform the design of the intervention, a lived experience design group was established. Participants were asked about their experiences of support following their suicide attempt, their needs during this time, and how these could be addressed in a brief contact eHealth intervention. The intervention design was also informed by consultation with lived experience panels external to the project and a clinical design group.

**Results:**

Prompt outreach following discharge, initial distraction activities with low cognitive demands, and ongoing support over an extended period were identified as structural requirements of the intervention. Key content areas identified included coping with distressing feelings, safety planning, emotional regulation and acceptance, coping with suicidal thoughts, connecting with others and interpersonal relationships, and managing alcohol consumption.

**Conclusions:**

The RAFT (Reconnecting AFTer a suicide attempt) text message brief contact intervention combines SMS contacts with additional Web-based brief therapeutic content targeting key risk factors. It has the potential to reduce the number of repeat suicidal episodes and to provide accessible, acceptable, and cost-effective support for individuals who may not otherwise seek face-to-face treatment. A pilot study to test the feasibility and acceptability of the RAFT intervention is underway.

## Introduction

Hospital-treated deliberate self-harm (DSH) is the single strongest risk factor for subsequent suicide, and repeat episodes and suicide are key clinical outcomes [1]. With a 1-year repetition rate of 15%, and 30% in those with a history of previous episodes [2,3], engaging with and treating self-harming patients soon after they present to the emergency department (ED) is important for reducing future suicidal behavior. The risk of repetition is highest in the first month after discharge; however, the risk remains elevated for at least 12 months [3].

Ongoing care after discharge is critical, and a failure to provide rapid and effective follow-up after DSH is associated with increased risk of further DSH, suicidal behavior, and death by suicide [4]. However, a data linkage study of 67,035 hospital-admitted DSH cases from 2005 to 2011 in the Australian state of New South Wales found that only 63% of patients received any mental health care within the public system following discharge [5], with only 41% having contact with a community mental health service in the month following discharge from an inpatient admission [6]. There is therefore a need to provide additional outreach and support following discharge.

A meta-analysis found that postdischarge brief contact interventions, such as letters, postcards, crisis cards, and telephone calls, were associated with a significant reduction in the number of repeat episodes per participant, although nonsignificant reductions were observed in the number of patients with any repeat attempt [7]. In addition to showing promising clinical outcomes, brief contact interventions overcome some of the barriers to implementation of more intensive forms of aftercare, including resource limitations and difficulty in engaging patients in ongoing treatment. Therapy-based interventions are generally expensive and require delivery by mental health professionals within traditional services, making them difficult to implement and potentially hard to access. Furthermore, a substantial number of patients are unable or unwilling to engage in face-to-face treatment but may be willing to engage with a lower intensity intervention.

There is increasing interest in the use of e-mental health interventions to overcome the barriers to traditional therapeutic interventions, including in the field of suicide prevention [8,9], but such interventions rarely support individuals following a suicide attempt [10]. There is, however, an increasing research focus on technology-supported brief contact interventions, and simple *caring contact* text messages (short message service, SMS) are currently being trialed [11,12]. We developed the RAFT (Reconnecting AFTer a suicide attempt) intervention to extend the scope of these text message contacts by including additional links to Web-based brief therapeutic content targeting proximal risk factors. We report on the development of the intervention, which was codesigned with lived experience groups and an expert panel and is designed for patients who have been recently discharged from the ED.

## Methods

Various frameworks have been proposed for participatory design and codesign of e-mental health interventions. Following the Patient-Clinician-Designer framework [13], we sought to engage those with lived experience of a suicide attempt, health care professionals, and designers throughout the design process (with the research team contributing expertise to the latter 2 stakeholder groups).

### Lived Experience Design Group

Recognizing the importance of lived experience in the implementation of this project, a lived experience design group was established to inform its design. The project-specific design group was recruited through the Black Dog Institute’s website and social media channels, through a research register of people who had previously consented to be contacted about research projects, as well as through partner organizations. Potential participants were asked to contact a member of the research team to screen for eligibility. Participants had a history of a suicide attempt, but not in the immediately preceding month, and were not currently experiencing severe suicidal ideation (if a current suicide plan, means, or intent was endorsed). Eligibility was initially based on an age of 18 to 25 years, which was later broadened to 18 to 65 years. Ethics approval for the design group was obtained through the University of New South Wales Human Research Ethics Committee (HC14272).

A total of 16 potential participants contacted the research team, expressing interest in the lived experience design group, 14 of whom were female. Five individuals were ineligible because of current severe suicidal ideation (n=4) or a suicide attempt within the previous month (n=1). Four individuals were eligible but could not attend a focus group because of scheduling constraints. The remaining 7 eligible individuals were scheduled to attend 2 focus groups (comprising 3 and 4 participants, respectively); however, 3 participants did not attend the second session, so this was conducted as a one-to-one interview with the one attending participant. All 4 design group participants were female.

Discussions were audio-recorded, then transcribed by a member of the research team. A second researcher reviewed the transcripts and the recordings to ensure accuracy. A member of the team then undertook thematic analysis of the transcripts, following Braun and Clarke [14], and developed an initial coding scheme. A second researcher then reviewed the coding, and any disagreements were resolved by discussion until consensus was achieved.

### Content Development

A clinical design group was also established for this project to capture a range of researcher and clinician perspectives. The 5 members of this group had expertise in e-mental health, clinical psychology, liaison psychiatry, emergency medicine, drug and alcohol services, and mental health epidemiology.

On the basis of the analysis of the focus group discussions, a high-level design incorporating the broad content areas and mode of delivery was created by the research team and presented to the clinical design team. Through a group-setting guided discussion, the following aspects of the intervention design were established: the relative importance and scheduling of the identified content areas, thematic and therapeutic connections between content areas, and low-intensity interventions or resources that can be provided for each content area.

### Design Refinement

Following the clinical design group discussion, the research team developed a comprehensive draft of all content (SMS text messages and Web-based content) within the intervention. The importance of continued engagement with people with lived experience was recognized, as were the challenges of recruiting and scheduling for the initial focus groups. Therefore, the existing Centre for Research Excellence in Suicide Prevention’s Lived Experience Committee (CRESP LEC) and the Black Dog Institute’s Lived Experience Advisory Panel (BDI LEAP) were approached. Members of these groups were invited to provide verbal or written feedback on the draft content as well as a number of look-and-feel options developed by a creative agency. These groups, along with clinical and research teams, included both women and men. Therefore, engagement with these groups also allowed a broader range of lived experience perspectives to be incorporated into the design process.

### Implementation and Testing

Following these rounds of consumer and clinical feedback, a prototype implementation of the RAFT intervention was developed. Members of the clinical design group and the CRESP LEC were invited to test the realized system, and verbal or written feedback was provided on the overall user experience and usability, as well as the content and its presentation. The system was iteratively updated in response to the feedback received.

## Results

### Design Process

Our first discussions with the lived experience design group focused on what help and support was available to participants following their suicide attempt, what was helpful and not helpful, what support they would have liked to have received, and the scope for incorporating such strategies into an electronic health (eHealth) intervention. During these initial discussions, the concept of follow-up by SMS text messages was supported as follows:

I think it would have been useful to me...I just needed any contact from anybody, and when I saw the system, and when I was told the system was supposed to respond in a certain way for 48 hours and they didn’t—it really hurt me.

P1

Compared with other forms of follow-up contact, SMS text messages were broadly preferred, as seen in the quote below:

Generally speaking I quite appreciate phone calls, but I think after a suicide attempt I perhaps wouldn’t have appreciated that so much.

P4

However, it was acknowledged that some may not find such contact helpful; one of the participant described how the contact may indeed be perceived as:

...personification of a health system that sees you as a problem instead of a person.

P2

When asked about what techniques participants had found helpful following their suicide attempt, distraction activities such as games, drawing and coloring were described. One of the participants stated:

...where you can just play games as a way to keep yourself, your mind busy. I did have Sudoku books, which I did a lot of. And I did a lot of those dot paintings with textas.

P3

Participants highlighted that these activities were typically of low-intensity, as expressed by one of the participants:

...not much energy either...[an activity] that is achievable.

P2

In addition to these short-term strategies, participants also identified longer-term support, which was helpful following their suicide attempt. One participant stated:

...any sort of approach which looks at acceptance, and I mean, emotion regulation was very important for me.

P4

Participants frequently described difficulties in communicating with friends and family following their suicide attempt:

My best friend at the time stopped talking to me for 3 weeks.

P2

I’ve been walked past as though I don’t exist by other family members.

P1

When asked about the scheduling of messages, participants indicated frequent messages would be useful, but not *too* frequent:

I think [the first message should be] the day after discharge.

P4

…maybe every 2-3 days as the default…I would say a maximum of a week because otherwise you’re not taking care of yourself enough.

P2

I’d say one a day, but that would probably get irritating too. So probably I’d do that for a few days, and then extend it.

P3

Although the possible duration of messaging was not directly discussed, participants described a general need for longer-term care as follows:

…our hospitals [are] very acute driven...and they do that very well, but I think with mental illness, you need to get at the chronic illness, and how you manage that on a longer timeframe...I think I may not have ever have got to the second [suicide attempt] if I had been cared for in a way that had a longer term vision.

P4

On the basis of the lived experience discussions described above, the following key topic areas were identified: initial distraction activities to cope with distressing feelings, emotional regulation and acceptance, and interpersonal relationships. The clinical design group expanded upon these areas to also include safety planning, as part of a best-practice safety protocol; coping with suicidal thoughts, as an extension of emotional acceptance; and managing alcohol use, as this is a proximal risk factor. The needs for prompt outreach following discharge, initial content with low cognitive demands, and ongoing support over an extended period were also endorsed. To match this trajectory, an initial contact within 24 hours was proposed, followed by weekly messages related to the topics identified above, then monthly reminders until 12 months.

The content of the SMS text messages and additional Web-based content related to the identified topics was then drafted, with feedback obtained from the clinical design group and the CRESP LEC. Two proposed user interface wireframes were designed, with feedback also obtained from the clinical design group, CRESP LEC and BDI LEAP. Following both sets of feedback, the specifications were finalized, and the realized system is described below.

### System Design

The aim of RAFT is to provide a text message–based follow-up intervention, combining regular SMS contacts and links to Web-based therapeutic content and resources focused on the 6 content areas. These key areas, identified through thematic analysis, include coping with distressing feelings, safety planning, emotional regulation and acceptance, coping with suicidal thoughts, connecting with others and interpersonal relationships, and managing alcohol consumption. The sequence of SMS text messages is intended to start with low cognitive demands for the early SMS text messages during the initial crisis period, with additional therapeutic components introduced later. Each SMS text message contains a brief message related to the content area, with a link to information and brief therapeutic content on the study website.

#### SMS Text Message Component

**Figure 1:**
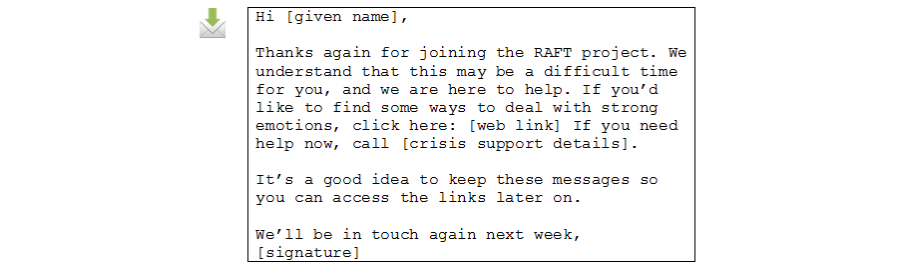
Example of the first text message (coping with distressing feelings). Customized text is shown in [square brackets].

Upon registration, users automatically receive a series of personalized text messages at a predefined schedule. The first text message (coping with distressing feelings) is sent within 24 hours of user registration, with messages related to the other topics sent weekly until week 6. Each message is customized with the recipient’s given name and signed by the team from their presenting hospital. Messages also express the treating clinician’s wish that the person is well and invite them to recontact their relevant local health service if needed. Each message also contains a unique link to the relevant Web content (Figure 1).

**Figure 2:**
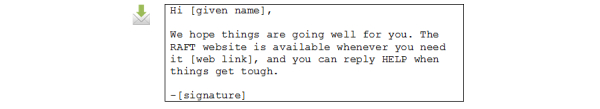
Example of a monthly reminder message. Customized text is shown in [square brackets].

Following the 6 weekly SMS text messages during this immediate postdischarge period, the participant then receives monthly reminder messages until just over 12 months have passed (Figure 2). The final message is delayed slightly to avoid the anniversary date of the index presentation. These messages contain reminders to revisit the Web-based content or to contact a crisis service or a health professional if required.

**Figure 3:**
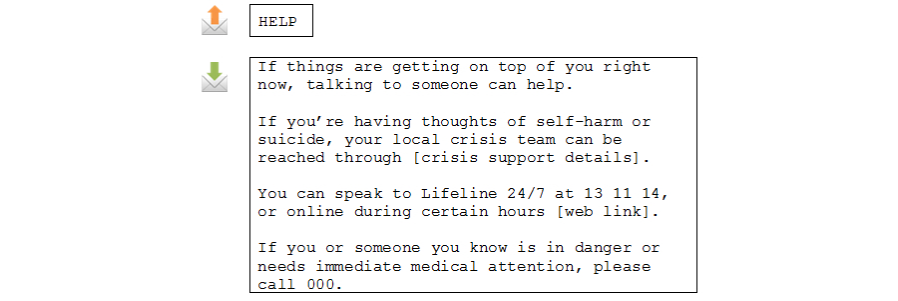
Example of automatic response to the HELP keyword, providing local crisis support, national crisis support, and emergency service details. Customized text is shown in [square brackets].

**Figure 4:**
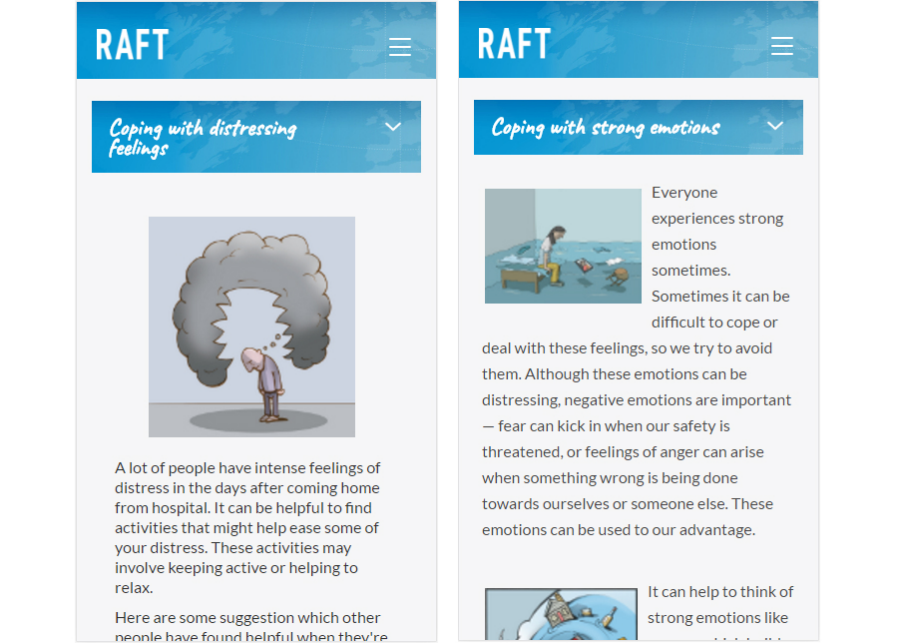
Sample screenshots from the webpages related to the first and third text messages. Left: “Coping with distressing feelings” on day 0. Right: Emotional regulation and acceptance (“Coping with strong emotions”) on day 14.

As part of the safety protocol, participants can reply to any message at any time with the keyword “HELP.” This triggers an automated response containing contact details of their local acute care team, the national Lifeline crisis telephone line, and if the participant feels in immediate danger, the emergency services (triple zero; Figure 3). The local acute care team is suggested as the first point-of-call to encourage appropriate triage to self-care or local health services. In addition to the “HELP” keyword, participants are able to opt out of further participation at any time by replying “STOP” to stop receiving future messages.

#### Web Component

If a user clicks on one of the links contained within the text messages, the relevant content within the study website is loaded. The content for each of the six content areas is described below. Figure 4 shows sample screenshots of the Web content.

##### Day 0: Coping With Distressing Feelings

This section aims to help participants cope with the distressing feelings present in the immediate period following discharge from hospital. It attempts to normalize feelings of distress and provides simple emotional regulation strategies, including distraction and calming activities. It offers strategies that members of the lived experience design group found helpful, including links to other free apps and resources, and includes listening to music, engaging with art, practicing mindfulness, playing games, and coloring. Participants are also able to enter their own activities and rate whether each activity is helpful for them.

##### Day 7: Safety Planning

One week post attempt, this section encourages participants to create a safety plan in case a crisis reemerges. The rationale for a safety plan is described by Stanley and Brown [15], who suggest that the participant can create one on their own or with help from a friend or family member. Links are provided to a safety planning app or a downloadable document. The participant is reminded that the activities they found useful from the previous section can be included in their safety plan.

##### Day 14: Emotional Regulation and Acceptance

After 2 weeks, additional brief therapeutic content is presented. This section is based on acceptance and commitment therapy and encourages learning acceptance of strong emotions and effective emotional regulation techniques. Links are provided to brief Web-based mindfulness audio recordings for when the participant feels distressed, while also balancing the need for active regulation: participants are again referred back to any activities they found useful from the first section.

##### Day 21: Coping With Suicidal Thoughts

After 3 weeks, the participant is introduced to cognitive strategies that may be useful if suicidal thoughts reemerge. Thought defusion strategies are suggested and presented as a case study tailored to the participant’s gender. These strategies include recognition of unhelpful or *bully* thoughts, observing and naming these thoughts, and questioning their accuracy and utility.

##### Day 28: Connecting With Others and Interpersonal Relationships

After 4 weeks, an additional case study is presented in relation to managing interpersonal relationships and solving relationship difficulties. Helpful and unhelpful communication styles, thinking styles, and interpersonal behaviors are highlighted, along with encouragement to apply these to the participant’s own relationships.

##### Day 35: Managing Alcohol Consumption

The final content area focuses on managing alcohol consumption, a proximal risk factor for suicidal behaviors. The section starts with the AUDIT-C screener [16], and if this indicates potentially harmful drinking behaviors, the participant is encouraged to visit the Healthier Drinking Choices website, a localized Australian version of the Down Your Drink brief intervention [17].

Each webpage becomes available when the corresponding SMS text message is sent. Participants can browse back through earlier sections related to any previous messages and are not required to view the content in sequence. To assess which content areas are most relevant, engagement with both the text messages and Web components of the intervention are automatically measured. Measures include the proportion of messages that fail to deliver; whether participants withdraw or opt out of future messages through the “STOP” keyword and at what point; which links are most frequently clicked and when; and which pages are viewed on the study website, how often, and when.

## Discussion

### Principal Findings

We have presented the consumer-informed development and design of the RAFT SMS-based brief contact intervention. RAFT extends existing SMS text message brief contact interventions [11,12] through the inclusion of links to Web-based brief therapeutic content that the user can choose to access anytime. These links cover a range of factors, including coping with distressing feelings, safety planning, emotional regulation and acceptance, coping with suicidal thoughts, connecting with others and interpersonal relationships, and managing alcohol consumption. The intervention has been developed with extensive consultation with lived experience groups. To assess the feasibility and acceptability of the RAFT brief contact intervention, a 12-month pilot study is currently underway at 3 participating emergency departments across Australia.

Our design process identified some similar themes as those identified by Cooper et al in their analysis of requirements for a brief contact intervention [18]. Common features include the need for proactive follow-up immediately following discharge from the ED, with messages of support and encouragement with relevant support contacts. Cooper et al also identified some uncertainty about the optimal contact intensity and duration. We did not, however, identify reservations about the use of mobile phones as a contact method. This may be because of increased mobile phone adoption in the intervening years. Also, our advertising material mentioned the development of an eHealth intervention, therefore the design group participants in this study may have been more willing to accept a mobile phone–based strategy. This work also complements that by Ranney et al, who codesigned a brief intervention in the ED setting for high-risk patients with symptoms of depression and recent history of peer-violence [19,20]. Both interventions have been designed by focusing on current coping strategies as a target for SMS text message follow-ups. However, the target populations are different, with Ranney excluding those who are acutely suicidal.

While following a Patient-Clinician-Designer framework to engage multiple stakeholders, our approach did not seek to bring those with lived experience in direct contact with health care professionals. This was intended to be sensitive to participants’ needs [13], where such crossover may have created a more intimidating environment or reduced participants’ openness to discuss their experiences with existing services. Others have, however, reported that such interactions can lead to the creation of new design ideas in the mental health setting [21,22].

The RAFT intervention has been designed to be readily accessible to a large proportion of the population and has the potential to readily scale to other clinical services and settings. Although designed for an Australian setting, the content and support services can be readily adapted for international settings. This brief intervention may also be more acceptable to a younger population and those who are unable or unwilling to receive face-to-face treatment.

### Limitations

A number of limitations are acknowledged in the development of the RAFT intervention. First, the project-specific lived experience design group only contained 4 participants and, therefore, it is unlikely that the experiences described represent the full range of lived experience. Furthermore, the group was exclusively female, possibly reflecting the gender disparity between suicide attempts and completed suicides, and additional targeted recruitment attempts were unsuccessful. Therefore, the acceptability for male participants is unclear and will be determined through the pilot study.

Our screening process only captured whether a participant was in the relevant age range for this formative research study, and we did not collect further demographic details. Additional data collection may have allowed further insights into the suitability of this intervention for specific age ranges; however, such data are not available. Individuals’ history of suicidal behavior may also have provided additional insights; however, we focused on coping strategies subsequent to their suicide attempt rather than the actual attempt or preceding events.

SMS text messaging may be considered a relatively simple or old technology, particularly when compared with smartphone apps. However, apps may encounter a higher barrier to adoption in the ED setting, with limited feasibility for downloading and installing an app during the routine discharge process. Text messaging is also available to a larger proportion of the population, including lower income participants who may not own a modern smartphone, although the Web-based content may not be fully accessible to these participants. The messaging infrastructure also allows for longer term automatic deployment with fewer ongoing maintenance and update requirements than an app.

The automated text messages proposed in this system may be perceived by some as an extension of a health care system that does not care, as described by one participant. Other participants, however, described that any follow-up would be appreciated, especially as personal follow-up is often promised but not always delivered. It may be possible to extend this automated SMS system with additional keyword responses; for example, the “HELP” message could trigger a follow-up phone call from a crisis service.

### Conclusions

We have developed a new SMS text message–based brief contact intervention, delivered over 12 months following an ED presentation for a suicide attempt, which expands previous caring contact interventions with additional Web-based brief therapeutic content. Such an approach has the potential to reduce the number of repeat episodes of suicidal behavior and to reach young people at risk of self-harm and suicide who are unable or unwilling to undergo face-to-face treatment with health professionals. Our pilot study aims to assess the acceptability and feasibility of delivering this intervention through an ED setting. Widespread mobile phone technology allows RAFT to be readily deployed at scale and is likely to be more acceptable to a younger target audience than alternative clinical therapeutic options.

## Abbreviations

BDI LEAP: Black Dog Institute’s Lived Experience Advisory Panel
CRESP LEC: Centre for Research Excellence in Suicide Prevention’s Lived Experience Committee
DSH: deliberate self-harm
ED: emergency department
RAFT: Reconnecting AFTer a suicide attempt
SMS: short message service
